# Death Rates among Detained Immigrants in the United States

**DOI:** 10.3390/ijerph121114414

**Published:** 2015-11-12

**Authors:** Megan Granski, Allen Keller, Homer Venters

**Affiliations:** Center for Health and Human Rights, NYU Langone Medical School, New York, NY 10016, USA; E-Mails: megan.granski@nyuymc.org (M.G.); Allen.Keller@nyumc.org (A.K.)

**Keywords:** immigration, detention, mortality, suicide

## Abstract

The United States system of immigrant detention centers has been the subject of considerable scrutiny with respect to health care of detainees. We sought to characterize the rates and types of deaths that have occurred within this system between the years 2003–2015. We analyzed a file of detainee deaths released by the U.S. Department of Homeland Security as part of a freedom of information request. Between 2003 and 2015, 150 deaths were recorded. During this time period, the annual rate of death among detainees dropped dramatically, whether measured by annual admissions or by person years of exposure. The most common causes of death were cardiovascular, cancer and suicide. More research is needed to adequately account for the contributors to these declining rates of death in immigration detention settings.

## 1. Introduction

Over the past ten years the media, governmental, and nongovernmental organizations have raised concerns about health care provided to detainees held in the custody of U.S. Immigration and Customs Enforcement (ICE), a department under the US Department of Homeland Security (DHS) [1,2,3]. These concerns have identified gaps in ICE health policies and procedures relating to initial screening of newly arrived detainees, the application of an emergency care approach to a population with high rates of chronic disease, and a lack of adequate testing, diagnosis and treatment for HIV and other treatable diseases. In addition, ICE has been criticized for diverting patients with mental illness into detention instead of allowing them to receive community-based treatment. However, relatively little has been published on trends in detainee deaths. In this paper we present data on the death rates among detainees in U.S. Immigration Detention from 2003 to 2015.

The United States detains noncitizen immigrants to guarantee court appearance for immigration proceedings. ICE is responsible for the oversight of medical care and conditions of detention in the three types of facilities in the United States where immigrants are detained. These include service processing centers, which are owned and operated by ICE; contract detention facilities, which are managed by private companies; and state and local jails that enter intergovernmental service agreements (IGSAs) with ICE. The ICE Health Service Corps (ISHC) is responsible for providing and coordinating health care services for detained immigrants. Over the past decade, ICE has worked to concentrate the number of detainees who are held in sites with ISHC staff on site. In addition, ICE has promulgated several rounds of new medical policies for detention settings, including in 2000, 2008 and 2011. Our experience in advocating for patients, visiting detention centers and meeting with ICE has been that health practices have improved for many detainees, however we sought to quantify changes in death rate among ICE detainees, and also to provide context for any changes we perceived.

## 2. Methods

Data for this analysis were obtained from the ICE freedom of information act (FOIA) library, which is publically accessible on the internet [4]. ICE posts documents released through FOIA requests and categorizes this information in broad categories such as Contracts, Staffing Plans and Testimony. Within the category of Reports, ICE posts a list of deaths. At the time of this analysis, the file was labeled “Detainee Deaths—October 2003 through 26 May 2015”. Because the 2003 and 2015 deaths did not represent a full calendar year, deaths from 1 January 2004 through 31 December 2014 were utilized for yearly rates, representing a total of 144 deaths. The entire file was then used to perform an analysis of deaths by year, as well as deaths by type of facility, which included deaths from October 2003 through May 2015, a total of 150 deaths. Causes of death were categorized by 2 physicians. Information about length of stay and average daily population were obtained from ICE and utilized to formulate rates of death in two ways: Deaths per person years and deaths per 100,000 admissions. No financial or other conflicts of interest exist for the authors.

## 3. Results

Overall, the yearly number of deaths occurring in ICE facilities has dropped dramatically between 2004 and 2014 (Table 1). In 2004, 32 deaths occurred in ICE facilities, with generally decreasing numbers until 2014 when 6 deaths were reported. During this time period, the average daily population increased from 21,928 in 2004 to a plateau of 33,000–34,000 in 2011 through 2014. Conversely, the average length of stay started at 40.4 days in 2004, but decreased to a plateau of approximately 30 days by 2008, which was sustained though 2014. The death rate based on person years of exposure was 0.146 deaths per 100 person years in 2004 and fell steadily throughout the time period to 0.018 in 2014. The death rate based on number of admissions was 16.15 per 100,000 admissions in 2004, steadily decreasing to 1.5 per 100,000 admissions in 2014. The greatest decreases in both calculated rates occurred in the years 2004–2007.

**Table 1:** Death Rates in U.S. Immigration and Customs Enforcement (ICE) Custody 2004–2014 (*n* = 144).

Year	Deaths	Average Daily Population (ADP)	Average Length of Stay (ALOS)	Admissions per Year = (365/Average Length) × ADP	Death Rate per Person Year = (Deaths/ADP) × 100	Death Rate per 100,000 Admissions= (Deaths × 100,000)/Admissions per Year
2004	32	21,928	40.4	198,111.88	0.146	16.15
2005	20	19,718	38.5	186,936.88	0.101	10.7
2006	18	22,975	33.7	248,839.02	0.078	7.23
2007	8	30,295	36.9	299,665.99	0.026	2.67
2008	16	31,662	30.5	378,905.90	0.051	4.22
2009	10	32,098	31.2	375,505.45	0.031	2.66
2010	9	30,885	32.1	351,184.58	0.029	2.56
2011	11	33,330	29.7	409,611.11	0.033	2.69
2012	5	34,260	27	463,144.44	0.015	1.08
2013	9	33,788	29.4	419,476.87	0.027	2.15
2014	6	33,227	30.4	398,942.60	0.018	1.5

When cause of deaths was categorized, the most common category was cardiovascular (33.3%), followed by cancer (16.1%), suicide (listed as suicide or asphyxia or self-inflicted injury, 13.4%) and other (12.8%, Table 2). Deaths included in the other category included natural causes, diabetes complications, lung disease, rabies, and nonspecific or unknown causes in 5 cases. The 5 cases attributed to the trauma/accident category included 2 electrocutions, and one each stabbing, traumatic brain injury and drowning. The two deaths categorized as overdose included one each methamphetamine and cocaine intoxication.

**Table 2:** Deaths by Cause (*n* = 150).

Category	Number	Percent
Overdose	2	1.33
Liver Disease	3	2.0
Seizure	3	2.0
Trauma/accident	5	3.3
Renal	6	4.1
HIV/AIDS complications	6	4.1
Infection	12	8.2
Other	19	12.8
Suicide	20	13.4
Cancer	24	16.1
Cardiovascular	50	33.3
Total	150	100.00

When facility type was analyzed for all 150 deaths, 54.5% of cases occurred in Inter-governmental Service Agreement (IGSA; dedicated or DIGSA; intergovernmental ) settings, while 15.2% occurred in Federal Bureau of Prisons (BOP) settings, 11.0% in Contract Detention Facilities (CDF), and 9.7% in service processing centers (SPC). Among the suicide cases, 16 out of 20, or 80%, occurred in IGSA settings. Of note, 5 of the suicides occurred in the Eloy Federal Contract Facility, while no other facility had more than one suicide. Both overdose deaths occurred in holding facilities.

## 4. Discussion

These data reflect a significant decline in the number of persons who have died in ICE custody over the past 11 years, as well a corresponding decrease in the rate of death, whether calculated by person years of exposure or number of admissions. The rate of decline in deaths in ICE custody exceeds any decline reported in jail or prison systems during a similar time [5,6] and may reflect changes in the profile of persons being detained as well as health and custody practices inside the ICE detention system. In terms of changes in ICE detention practices, ICE statistics point to two important demographic changes over the past decade. First, DHS has dramatically increased the use of expedited removal, a process whereby persons apprehended near the border agree to be quickly removed to their country of origin, relinquishing the right to appear before a judge and to apply for status in the U.S. Between 2005 and 2013, the use of expedited removal doubled, resulting in a dramatic increase in the share of all persons who come into contact with ICE who are quickly returned to their country of origin [7]. Many of these people may not enter an ICE facility and may have contact only with the U.S. Border Patrol or ICE office staff. Statistics about deaths in Border Patrol Custody as well as deaths during the border crossing and after removal via expedited release were not available for this analysis, but they represent a potential shifting of mortality from ICE detention.

A second demographic shift among ICE detainees may be linked to the increasing reliance on programs such as the Secure Communities program and the Priority Enforcement Program that direct immigrants arrested for crimes into ICE custody from jails and prisons. These programs rely on coordination between local criminal justice systems and ICE, so that persons who enter custody for a criminal charge may be discharged directly into ICE custody. From a health standpoint, this arrangement may result in local jails doing much of the initial screening and treatment of ill patients and some of the highest risk patients may either have died while in criminal justice detention or been diverted away from ICE detention. Accordingly, persons arriving in ICE custody through this path may be lower risk than others who are detained directly from the community, especially as relates to common causes of early admission death such as alcohol withdrawal and untreated seizure disorder. Since 2008, ICE deported more than 283,000 convicted immigrants as a result of Secure Communities. The percentage of detainees identified through the Secure Communities program grew from 4% in FY 2008 [8,9] to 21% in FY 2013 [9,10]. Individuals transferred to ICE via the Secure Communities program generally have received recent screenings and medical care in jail or prison, which may contribute to the decrease in mortality.

Another potential contributor to decreasing mortality in ICE detention is improvement in ICE medical policies and practices. ICE introduced a set of performance based national detention standards for all detention facilities in 2008, and again in 2011. ICE implemented these standards with considerable exemptions in IGSA settings because of the small scale and lower level of resources in many of these county jails. Over time, ICE has both increased the compliance demands on IGSA’s and also increased the utilization of U.S. Public Health staff to provide direct care. These staff receive specialized training in delivering care to immigrants and they now number over 900, providing care to almost half of detainees (15,000) while also involved in oversight of care for the remainder (17,000) of detainees. Because we were unable to obtain the yearly breakdowns of detainee census in ISGA *versus* other facilities, we do not have a direct measure of the change in IGSA death rates. Rather, we can only note that over the time of this analysis, fewer detainees are being held in IGSA settings, where health services are less robust. From a health standpoint, housing fewer persons in small county jails and increasing the utilization of dedicated ICE facilities, which have health staff provided by the U.S. Public Health Service, may account for some amount of mortality reduction.

The causes of death described in this analysis are similar to those reported in U.S. jails [5,11]. The predominance of cardiovascular causes of death mirrors what is seen in the community and other detention settings. In terms of the causes of death, one striking aspect is the lack of death from alcohol withdrawal, a relatively common (though preventable) cause of death in the first 72 h of incarceration. The concentration of suicides in a single facility merits particular attention, as suicide screening and prevention is a shared responsibility between health and custody staff. Reporting by advocacy organizations has highlighted suicide prevention and sexual harassment investigation as lacking in private contract settings [12].

## 5. Conclusions

Overall, the rate of death in ICE detention settings has declined significantly. It appears as though suicide may be overrepresented as a cause of death in IGSA settings. Important limitations exist in this analysis, including the lack of information about mortality among persons who are briefly detained and removed outside of the ICE detention system. More analysis is needed to provide reliable interpretations of the reasons for these decreases in mortality rates among detained immigrants. In particular, more analysis is needed to quantify rates of death among those briefly detained by the U.S. Border Patrol, but not ICE, as well as those who have been removed to other nations and die shortly after arrival. Finally, because an increasing share of those who ultimately come into ICE custody start in criminal justice detention, more focused analysis of deaths in those settings could be undertaken.
